# Thin Film Composite Membrane for Oily Waste Water Treatment: Recent Advances and Challenges

**DOI:** 10.3390/membranes8040086

**Published:** 2018-09-21

**Authors:** Nor Akalili Ahmad, Pei Sean Goh, Zulhairun Abdul Karim, Ahmad Fauzi Ismail

**Affiliations:** Advanced Membrane Technology Research Centre (AMTEC), School of Chemical and Energy Engineering, Faculty of Engineering, Universiti Teknologi Malayisa, Johor 81310, Malaysia; nor_akalili@yahoo.com (N.A.A.); zulhairun@petroleum.utm.my (Z.A.K.)

**Keywords:** thin film composite, oily wastewater, forward osmosis, reverse osmosis

## Abstract

Oily wastewater discharge from various industry processes and activities have caused dramatic impacts on the human and environment. Treatment of oily wastewater using membrane technology has gained worldwide attention due to its efficiency in removing the amount and concentration of oil and grease as well as other specific pollutants in order to be reused or to fulfill stringent discharge standard. The application of thin film composite (TFC) membrane in reverse osmosis (RO) and forward osmosis (FO) for oily wastewater treatment is an emerging and exciting alternative in this field. This review presents the recent and distinctive development of TFC membranes to address the issues related to oily wastewater treatment. The recent advances in terms of TFC membrane design and separation performance evaluation are reviewed. This article aims to provide useful information and strategies, in both scientific knowledge advancement and practical implementation point of view, for the application TFC membrane for oily wastewater treatment.

## 1. Introduction

Pollution by oily wastewater, which is also known as produced water, has become one of the major worldwide issues as the consequences of rapid industrialization in oil and gas, petrochemical and metallurgical industries [1]. Serious oil spill accidents due to oil exploration and transportation has also further exacerbated the problem [2]. Other than oil and grease, typical oily wastewater also contains highly toxic substances, hydrocarbon compounds, heavy metals and suspended solid particles. Hence, the generation, discharge and leakage of oily waste from these industries have severely threatened public health and affected the marine ecosystem [3]. Additionally, the presence of wastewater has also caused the deterioration of crop production and devastation of natural landscape [4]. With the booming of oil and gas production, the water community has urgently called for promising approaches for oily wastewater treatment. As the management of oily wastewater has imposed considerable costs and challenges to the oil and gas industry, there is an urgent need to explore more cost-effective and energy-efficient technique to treat oily wastewater.

Among the wide range of contaminants found in oily wastewater, the separation of oil and grease presents the most challenge task. These oil constituents exist in the form of dispersed oil with size ranges from 20 to 150 μm, stable emulsified oil with droplet size <20 μm and free-floating oil with size >150 μm [5]. Commonly, oily wastewater has been treated in a number of conventional processes that involve the separation of oil/water emulsion, which is mostly based on physical processes such as adsorption, flotation, and coagulation [6,7]. Adsorption has been commonly used to treat oily wastewater as it is capable of removing most of the contaminants and result in nearly complete product water recovery. With the usage of high surface area adsorbent such as zeolite and organ clays, the usage of chemicals during this process can be minimized [8]. One of the most detrimental disadvantages of this method is the saturation of adsorbents by the highly concentrated waste hence the adsorbents must be disposed or regenerated after a relatively short duration of operation. The additional cost involved in the replacement and regeneration has made it unfavorable for long-term operation [9].

Flotation involves the addition of a floatation agent to assist in the adherences of oil particles to form colloidal particles and suspended in the water [10,11]. The oil suspension with lower density can then be easily removed from the wastewater. Flotation has been conventionally used due to its high processing capacity and separation efficiency despite some significant disadvantages such as high-energy consumption. Another commonly used method to treat oily wastewater is coagulation, which is known to be efficient in removing emulsified and dissolved oil, as well as some non-biodegradable organic compounds that co-exist in the wastewater [12]. However, coagulation also suffers from a number of drawbacks such as high treatment cost, long operation time, and the production of sludge. Recently, there has been increasing interest in advancing floatation and coagulation using electrochemical methods in order to counter the limitations of these conventional methods in terms of energy usage, stability, and production of waste residues [13,14]. Nevertheless, the efficiency of electrocoagulation and electroflotation is not only highly dependent on the nature of treated oily wastewater, but also more critically relying on the design and parameters of the electrochemical system such as voltage and current, types and properties of anodes, as well as the alignment of electrodes. 

Compared to the existing conventional methods that are relatively less efficient and require more complicated operations, membrane processes have been acknowledged as a simple and promising method to treat oily wastewater, particularly for the removal of oil droplets below 10 μm [15,16]. Membrane technology has been proven to be more effective not only in the removal efficiency, but also in other critical aspects such as energy efficiency, smaller footprint, as well as ease of installation, operation, and scaling up [17]. Typically, membrane processes for oily wastewater treatment are classified based on their pore sizes and the operating pressure. Microfiltration (MF), ultrafiltration (UF), nanofiltration (NF), and reverse osmosis (RO) are conventionally used pressure driven membrane processes [18]. Numerous studies have been performed to evaluate the effectiveness of these membrane processes to treat oil water emulsion. Recently, emerging membrane processes such as forward osmosis (FO) and membrane distillation (MD) have also gained attention for more sustainable oily wastewater treatment. The former osmotically-driven process exhibits energy efficiency and lower fouling propensity in the absence of hydraulic pressure [19,20], and meanwhile, the latter thermal-driven process is known as a green technology where low-grade waste heat can be harnessed for its operation [21]. The main driving factor for implementing FO and MD system versus the conventional pressure-driven system is associated to the energy consumption and capital expenses [22]. Nevertheless, the large-scale application is impeded by the challenge in draw solution recovery for FO and membrane wetting for MD. 

Membrane is the heart of the membrane processes that dictates the overall separation performance in treating oily wastewater. Membrane structure is one of the main factors determining the separation characteristics and transport mechanisms across the membrane. Owing to its relatively simple fabrication approach, which normally only involves single step flat sheet casting or hollow fiber spinning, integrally asymmetric polymeric membranes have been widely used in many membrane processes for decades [23]. Nevertheless, since the introduction of the first polyamide thin film composite (TFC) membrane, this new form of membrane has gained tremendous attention in research and industrial sectors due to the capability of TFC membrane in achieving the right combination of flux and salt rejection [24,25,26]. Another attractive advantage of TFC membrane is associated to the flexibility in selecting and optimizing the materials used for the formation of a micro-porous substrate layer and the selective thin film layer. Currently, TFC membranes have been widely applied in NF, RO, and FO mainly for seawater and brackish water desalination [27,28]. Due to the complex nature of oily wastewater that tends to increase the fouling potential of membrane during high hydraulic pressure operation, TFC membranes have been more commonly applied in FO to treat oily wastewater. Over the past few years, with the increasing use of membrane technology to treat oily wastewater, the innovative development of high performance TFC membrane for this purpose is also at the forefront of the water research community.

The need for treating oily wastewater using various strategies has been reflected in the increasing publication of review articles addressing the issues in this field. A comprehensive review on various innovative technologies used for oily wastewater treatment has been presented by Jamaly et al. [4]. Specifically, Zhu et al. reviewed the development of advanced polymeric and inorganic membranes as well as the nanocomposite membranes for emulsified oil/water mixture separation [29]. On the other hand, a recent review by Al-anzi and Ong focused on the progresses of nanomaterials and carbon-based nanocomposite membrane for the separation of oil/water emulsion [30]. Despite the efforts made on this topic, an overview of the development of TFC membrane for oily wastewater treatment still does not exist. As such, this review article presents the recent development of TFC for oily waste-water treatment. Firstly, the membrane processes that are feasibly used for oily wastewater treatment are reviewed. Next, the overview of the fabrication and characteristics of TFC membranes for the separation of oil/water emulsion is presented. Subsequently, the advances made in the development of high performance TFC membrane based on the selection of suitable materials and the formation mechanisms are highlighted based on the recent achievements made in the treatment of oily wastewater. Finally, the challenges and future outlook of this field are also reviewed before the conclusion is drawn.

## 2. Membrane Technology for Oily Wastewater Treatment

### 2.1. Current Membrane Technologies for Oily Wastewater Treatment

The oily wastewater discharge from industries such as metallurgical, transportation, petrochemical, and petroleum refineries has cumulatively contributed to the significant amount of waste to the water body. The typical oily wastewater normally consists of 50–1000 mg/L of oil and grease contents, depending on the nature and origins of the crude oil as well as their demulsification efficiency in the water. The conventional disposal practices have led to serious environmental pollution that has consequently posed severe hazards and threats to the aquatic ecosystem. Hence, treatment and removal of oily substances has become a critical aspect for the pollution control. Generally, the total oil and grease found in the oily wastewater discharged should be controlled in the range of 10–15 mg/L based on the environmental regulations set by most of the countries [31]. Unfortunately, traditionally applied approaches for oily wastewater treatment have been found inadequate to fulfill the requirement of various process industries to comply the discharge and reuse standards. In the recent decade, membrane technology has been recognized as an attractive alternative for oily wastewater treatment due to their efficiency in removing small oil droplets and offer treated water with a higher quality for reuse [16]. For years, polymeric and ceramic membranes have been feasibly applied in various membrane processes to treat oily wastewater [32,33,34,35]. Depending on the size, charge, and other water chemistries of the discharge components, membrane technologies with different driving forces have been used for the oil-water separation. Table 1 compares the advantages and limitations of various membrane technologies for oil/water emulsion separation.

MF and UF are two commonly applied membrane processes to separate the oil/water mixture. Despite the advantage of high water flux, asymmetric MF, and UF membranes that consist of relatively loose and large pores are inadequate to effectively reject smaller and stable emulsified oil particles [40]. Current advancement in UF for oily wastewater mainly focuses on the design of anti-fouling membranes through the accomplishment of novel strategies that involve the use of nanomaterials [41,42] and other functional materials [43]. Some of the desired properties that can be rendered by nanomaterials for oily wastewater treatment are hydrophilicity, high flux, and anti-fouling properties [44]. The development of photocatalytic UF membrane has also provided an attractive solution to minimize the impact of membrane fouling. Photo degradation and filtration can take place simultaneously in this integrated system to offer efficient oil/water separation, while photo degrading the organic molecules that could potentially foul the membrane surface [45].

RO is a very promising separation technology to yield high quality treated water owing to the almost complete rejection of most of the components found in oily wastewater. Over the past decade, a considerable amount of studies has evidenced the possibility of using RO to treat produce water [46,47]. RO has been prevailingly used for produced water desalination to generate fresh water to be reused at the oil and gas industries. However, the accumulation of oil and other substances such as dissolved and dispersed hydrocarbon, clay particles, surfactant, and salts on the surface of membranes has resulted in membrane fouling that consequently leads to flux deterioration [48]. With the increasing operating pressure and duration, the oil deposition exaggerates the formation of a continuous layer on the membrane surface [17]. Membrane cleaning and replacement is required where additional chemical and energy cost as well as extra downtime of the treatment installation are inevitably imposed to the process. Besides that, oil droplets and other contaminant molecules also reduce the effective pore size by forming a liquid liming within the pores. When this phenomenon takes place, not only the water flux is significantly affected, the membrane cleaning efficiency is also very effected. Due to the high susceptibility towards oil and hydrocarbon fouling, RO process normally requires effective pretreatment for oil and grease removal. In fact, the oil and grease contents in the RO feed should be reduced to <0.1 mg/L, as recommended by most of the membrane manufacturers [47]. 

One attractive alternative of conventionally pressure driven processes is FO. Compared to commonly used UF, this osmotically driven process is less energy intensive, while demonstrating better oil rejection and lower fouling tendency. In order to be competitively used in both bench and industry scales, the challenges deal with the membrane performance and economic viability of draw solution regeneration must be resolved. Currently, the FO membrane improvement is focused on the design of TFC and thin film nanocomposite (TFN) with desired characteristics. As a rule of thumb, a technological feasible draw solution should allow high water flux and low reverse salt flux to minimize the salt leakage as being easily recovered to improve energy efficiency [49]. Currently, a broad range of draw solutions with a promising FO performance and easy regeneration features has been explored. Some of the widely used draw solutions include low cost organic and inorganic salts with high osmotic pressure potential such as ammonium bicarbonate and polyelectrolyte [50]. Ge et al. explored the potentials of inorganic salt draw solutions that consists of oxalic acid ligand modified ferric and chromic complexes in FO process for oily wastewater treatment [51]. The high water solubility and the presence of ionic species with multiple charges upon the dissociation of the oxalic acid complexes that have favored the FO process. Owing to the structural uniqueness of oxalic acid which carrying abundant free terminal oxygen atoms to form a large network of hydrogen bonding with the surrounding water molecules, the reverse salt flux was greatly minimized. The treatment of 500 ppm oily wastewater using 1.0 M ammonium salt, which has been modified with the oxalic acid and chromic complexes, showed high oil rejection of 99.5% and negligible specific solute fluxes (Js/Jw) of 0.01 g/L.

While the performance of a single membrane separation process is sometimes unsatisfactory to meet the desired discharge or reuse requirement, hybrid membrane processes have been proposed as a viable mean to improve the quality of treated water. The hybrid system typically refers to the coupling of membrane technology with one or more other process units, which can also be a membrane technology or conventional treatment processes such as coagulation and absorption [52,53]. These processes are integrated to complement each other and offer solutions to tackle the limitation of a single membrane system. In the integrated system that consists of several stages of membrane processes, loose membranes such as UF and MF are used in the pretreatment unit to remove large oil droplets before subjected to more selective RO or MD processes [21,54]. While trace amounts of oil can still be detected in the permeate of MF and UF processes, RO and MD worked as an enhanced treatment process to further remove the small oil droplets in order to achieve almost complete oil removal and obtain high-quality water. 

A recently developed FO-RO pilot system for produced water treatment has evidenced the production of high quality permeate [55]. In this hybrid system, commercial cellulose triacetate spiral-wound FO membranes were used as pretreatment unit of raw produced water. The diluted draw solution was re-concentrated by the downstream RO system and returned to the FO system in a closed loop. Rejection of above 99% was observed for the major components found in the produced water such as salt ions, n-alkanes and fatty acids. Integrated UF-FO-MD system has also been reported to treat oily wastewater [56]. The osmotic pressure difference and vapor pressure difference rendered by high salinity and temperature oily wastewater was used as the driving forces of the FO process and MD process, respectively [51]. Further treatment using MD also allows the continuous regeneration and reuse of the draw solution. Such an integrated system can be promisingly utilized to realize simultaneous oil recovery and energy utilization while achieving high quality water regeneration [57].

### 2.2. Membrane Fouling in Oil-Water Emulsion Separation System

One of the most detrimental issues related to pressure-driven membrane processes, particularly reverse osmosis, is membrane fouling. During the treatment of emulsified oil/water mixtures, the accumulation of oil droplet, particularly those with sizes smaller than 20 µm, has resulted in severe fouling and a posed threat to the integrity of conventional membrane. Fouling of membrane by oil droplet is a complex phenomenon controlled by many key factors such as surface chemistry, structure, and charges [58]. Besides the many efforts devoted on designing superoleophobic–superhydrophilic membrane surface to reduce the fouling propensity, fundamental and mechanistic investigations on the stages of oil droplet deposition on the membrane surface, the structure and formation dynamics of the oil layer as well as the effects of hydrodynamic conditions on fouling reversibility during the separation of emulsified oil have also been performed [59]. The understanding in these aspects can foster the development of innovative membranes and the establishment of operational approaches to minimize fouling and enhance the sustainability of the membranes. 

It is known that oil droplets can deform, break-up, and coalesce flexibly to affect the fouling characteristics. The fouling mechanisms of membranes by oil have been comprehensively reviewed by Huang et al. [58]. In brief, three characteristic stages of membrane fouling have been identified i.e., (i) droplet attachment and clustering, (ii) droplet deformation, and (iii) droplet coalescence [60]. In the first stage, the oil droplets transport to the membrane surface in a long range and initiate adhesion of to one another. The second stage event takes place as a result from the attractive force between droplets to adjust the droplet- droplet and droplet-membrane contact lines. The kinetic energy of the attached droplets plays significant role in governing the dynamic of this three-phase system [61]. Out of these stages, droplet coalescence is known to be the most critical stage, which can dictate the severity of membrane fouling. In general, oil droplets with a relatively larger size tend to coalescent with other attached droplets to achieve a critical size. On the other hand, stable oil droplets with size smaller than 10 µm preferably remain stable as clusters on the membrane surface. Coalescence of oil droplet can also be intensified with the increasing permeate drag due to the prolonged residence time of oil droplets on the membrane surface [61]. 

Fux and Ramon investigated the dynamics of the oil-water-membrane system by observing the deposition, deformation, and detachment of stable oil droplet [62]. Their studies verified that membrane fouling by oil droplet is not solely determined by the oil droplet deposition, but also governed by the extent of droplet deformation. The hydrodynamic of the process such as permeate flux is one of the main parameters to control oil droplet rejection and fouling phenomenon. With the increasing permeate flux, the deformation takes place from a perfect sphere to hemisphere, leading to irreversible deposition that cannot be easily removed by cross-flow cleaning. Surfactant has been added intentionally to reduce oil/water interfacial surface tension and to prevent oil droplets from coalescing. It is known that surfactant influences the membrane fouling in multiple ways as it can alter the wetting behavior of oil droplets on the membrane by modifying the hydrophilicity/oleophilicity, as well as unfavorably entering the membrane pore and increasing the resistance to water permeation. The shape and charge of the surfactant head can significantly affect the fouling tendency. The latest study by Tanis-Kanbur et al. evidenced that the highest critical flux was obtained in the absence of surfactant stabilization due to the stronger oil-water interaction over oil-membrane interaction, hence the oil droplets remain in the bulk aqueous feed [63]. When the oil emulsion was stabilized with cationic surfactant dodecyltrimethylammonium bromide (DTAB), the lowest critical flux was achieved as the weak oil–oil repulsion has resulted in the extensive coalescence of the oil droplets.

## 3. Thin Film Composite Membranes for Oily Wastewater Treatment

**Figure 1:**
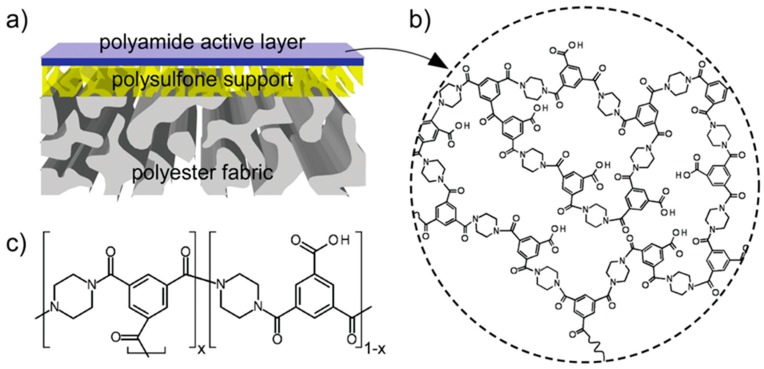
(**a**) Schematic diagram of the structure of typical TFC membrane, (**b**) the crosslinked structure of polyamide layer formed via interfacial polymerization (**c**) structural formula of trimesoyl chloride and piperazine monomers used in the formation of polyamide, with copyright permission from [64], © 2013, John Wiley and Sons.

As shown in Figure 1, a typical TFC membrane structure consists of three layers, i.e., a thin selective layer, an intermediate polymeric porous support and an optional nonwoven polyester fabric [64]. The polymeric support layer is usually made of semi-hydrophobic polymer such as polysulfone (PSF) and polyethersulfone (PES). These polymeric materials are cheap, have good chemical and mechanical stability and most importantly they show excellent membrane formability via a simple phase inversion technique. The performance of the polymeric substrate layer in TFC membrane is primarily based on the dope formulation and fabrication conditions, which is similar to those integral asymmetric membranes. A desired TFC membrane should have a sponge-like structure on top of substrate layer and finger-like structure throughout the membrane to ease the water transportation [65]. Many studies have been performed to obtain optimized conditions to yield stable porous support membrane to take account for both water permeability and mechanical strength. The selective layer of TFC membrane is made from a dense and cross-linked polyamide layer that can allow the fast water transport but hinder the passage of solute molecules [66]. The selective layer of TFC membrane can be fabricated based on several techniques including solution casting, in-situ graft polymerization, and interfacial polymerization. While there are many routes feasible to the thin film formation, interfacial polymerization is the most widely applied technique to form the polyamide selective layer. Since the breakthrough made in the formation of cross-linked aromatic polyamide TFC membranes and the subsequent introduction into the market, the exploration of new monomer materials for TFC selective layer has declined dramatically. Most of the research and development efforts are diverted on the optimization of the polymerization reaction through parametric studies of reaction time, curing time, and temperature as well as the composition and concentration of monomers and additives [67].

Compared to accustomed asymmetric membranes such as cellulose triacetate (CTA), TFC membranes have been proven to promote water permeation. Additionally, the high rejection nature of dense polyamide rejection layer ensures oil rejection of >99%, hence high water recover and good quality of treated water can be obtained from the oil-water separation. However, TFC is more susceptible to fouling and chlorine attack due to the vulnerable characteristic of the polyamide active layer [68,69]. Additionally, the higher fouling propensity was also attributed to the greater surface roughness and higher initial water flux [70]. The strong hydrogen bonding capability due to the presence of carboxyl groups (-COOH) found on the TFC active layers has further worsened the fouling as stronger adhesion force is established at the membrane-foulant interface [71]. 

**Figure 2:**
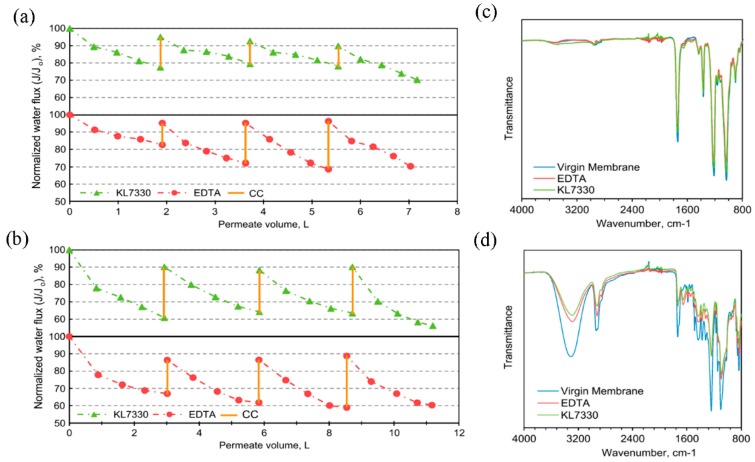
Normalized water flux data of (**a**) the CTA membrane and (**b**) the TFC membrane that were chemically cleaned with KL7330 and EDTA. ATR-FTIR transmittance spectra of (**c**) CTA and (**d**) TFC after the cleaning with KL7330 and EDTA, with copyright permission from [72], © 2015, Elsevier.

Coday et al. performed a comparative study to assess the produce water treatment performance of asymmetric CTA membrane and surface modified polyamide-based TFC membranes manufactured by Hydration Technology Innovations (HTI) [72]. In general, all tested membranes demonstrated excellent rejection of dissolved ions and organic compounds. Under the same operating conditions, the non-commercial surface modified TFC membrane exhibited water permeability of 1.04 L/m^2^·h·bar, which was more than 200% higher than that of CTA membrane with similar structural parameters. Similar observation has been made in a recent study conducted by Bell et al. where commercial CTA and TFC were used to treat produced water in the FO mode and 1 M NaCl was used as draw solution [73]. The osmotic backwashing of the membranes using KL7330 and EDTA showed that the flux decline was near linear for both membranes (Figure 2a), indicating that the fouling phenomenon resulted by the components in produce water was primarily due to the foulant–foulant interaction that took place at the cake layer surface. It was interesting to find that upon the chemically assisted backwashing, the smooth surface of the modified TFC has enabled better water flux recovery of 73% as compared to CTA. However, a membrane-fouling autopsy, using techniques such as ATR-FTIR and zeta potential, has suggested that the polymer chemistry of TFC was highly sensitive towards the chemical interaction with contaminants in the produced water. As a result, significant shift in the ATF-FTIR peak was observed in Figure 2d. In fact, the physical and chemical damages of TFC membranes due to some inevitable factors such as increase in differential pressure, oxidation, backwashing pressure as well as scaling and fouling have been commonly reported in many previous studies.

For osmotically driven membrane processes such as FO, one of the most significant challenges is the occurrence of internal concentration polarization (ICP) as a consequence of the dilution of draw solutes or the accumulation of feed solutes in the support layer [74]. The former condition is known as dilutive ICP, whereas the latter is known as concentrative ICP. Due to the asymmetrical structure of TFC membrane, FO can be operated in two modes, i.e., AL-FS orientation where the active layer faces the feed solution and AL-DS orientation where the active layer is faced against the draw solution. In the Al-FS orientation, the dilutive ICP occurs within the membrane substrate layer during the transport of water from feed solution to draw solution. On the other hand, AL-DS orientation causes concentrative ICP due to the accumulations of solute within the polymeric substrate [75]. The pore clogging will in turn compromise the water flux and heighten the fouling of membrane. The deterioration of membrane performance is exacerbated especially when dealing with highly viscous and concentrated oily wastewater. Therefore, for oily wastewater treatment based on FO operation, a relatively new concept of double skinned membrane has been established [76,77,78]. 

**Figure 3:**
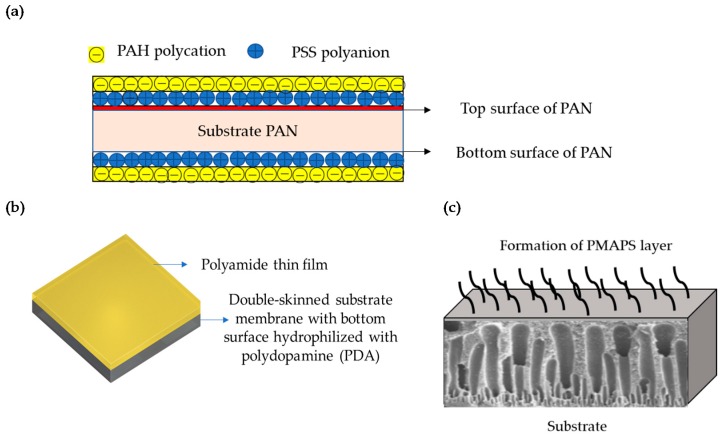
The formation of the secondary selective layer in double-skinned through (**a**) layer by layer of PAH and PSS, with copyright permission from [79], © 2012, Elsevier; (**b**) hydrophilization of PDA, with copyright permission from [74], © 2016, Elsevier; and (**c**) grafting of PMAPS [77].

The double skinned membrane can be described as a sandwiched structure with two selective layers at the top to and bottom parts of the polymeric substrates. While the primary polyamide selective layer has been feasibly formed via interfacial polymerization, the formation of secondary layer can be tailored by introducing additional functionality at the bottom part of the substrate through techniques such as layer by layer assembly [79], hydrophilization [74], and grafting [77]. As shown in Figure 3a, the secondary selective layer was formed by assembling positively charged poly(allylamine hydrochloride) (PAH) and negatively charged poly(sodium 4-styrene-sulfonate) (PSS) polyelectrolytes in an alternate manner. In Figure 3b, the mussel inspired polydopamine (PDA) rejection layer was formed through surface hydrophilization, whereas Figure 3c illustrates the grafting of zwitterionic polyelectrolyte brush (poly(3-(*N*-2-methacryloxyethyl-*N*,*N*-dimethyl) ammonatopropanesultone) (PMAPS). The double skinned membrane allows the operation to be performed with the polyamide layer facing the draw solution so that higher intrinsic flux, lower internal concentration polarization, and fouling propensity can be achieved. Interestingly, these bottom selective layers can be custom designed by selecting materials that render surface hydrophilicity and surface charges as well as chlorine resistant and anti-fouling properties.

Currently, major focus of the research in membrane technology for oily wastewater treatment is related to the establishment of various membrane antifouling strategies such as nanomaterial incorporation [29,80], surface hydrophilization [81], zwitterionic coating [82], photocatalytic decomposition [83], and electrically enhanced or switchable fouling resistant [84]. Zwitterionic polymers such as 4-Bis(3-aminopropyl)-piperazine propane carboxylate (DAPPC), *N*,*N*-diethylethylenediamine (DEDA) with excellent hydrophilic, oleophobicity and self-cleaning behavior have been used for TFC membrane modification to improve the water flux and antifouling behavior [85]. Excellent anti-biofouling feature has been observed on the PDA surface owing to the presence of catechol and amino groups that can initiate secondary reaction with any substrate for further modification [86]. Due to its excellent hydrophilicity and adhesive properties, PDA has been feasibly coated on a variety of polymeric surfaces via simple dipping in an aqueous solution to minimize the fouling resulted from protein and oil emulsions [87]. In a recent work by Yao et al., PDA and zwitterionic polymer were co-deposited to enhance the stability and anti-biofouling of polypropylene MF membranes [88]. The composite was stably anchored onto the membrane surface through a cross-linking reaction in alkaline environment. The formation of biofilm was effectively hindered due to the durable anti-bacterial activity of the coated layer against both E.coli and S.aures. Similar effect was also observed from the surface grafted with chitosan, a natural hydrophilic co-polymer endowed with abundant amino and hydroxyl groups. Chitosan and its water-soluble derivatives such as quaternary ammonium chitosan and carboxylated chitosan are known to effectively interact and breakdown the bacterial cell membrane [89]. 

Recent advances in the nanotechnology has promoted the application of nanomaterials in enhancing the characteristics of membrane. One of the most studied configuration of TFN consists of nanomaterials embedded within the polymer matrix or/and the polymeric substrate. In this nano-enabled membranes, the nanomaterials such as metal oxide nanoparticles, zeolites, carbon nanotubes (CNTs), and graphene family nanomaterials are commonly introduced to address some of the intrinsic limitations of conventionally developed TFC [90,91,92,93,94]. During the fabrication, the nanomaterials can be either added to the monomers during the interfacial polymerization of polyamide selective layer or/and physically mixed into the polymer dope of substrate.

## 4. Advances of Thin Film Composite Membrane for Oily Wastewater Treatment

Modifications of TFC polyamide layer and polymeric substrate have been performed to enhance the separation properties of the modified TFC membranes. Table 2 summarizes the modification strategies and the performances of the modified TFC membranes for oily waste water treatment. The tabulation indicates that the recent progresses of TFC membrane for oily wastewater treatment are mainly focused on the application of FO processes. Zhang et al. fabricated FO TFC membranes by grafting amine-terminated sulfonated poly(arylene ether sulfone) (NH2-BPSH) onto the polyamide layer for oily wastewater treatment. The in-situ grafting was facilely carried out based on the chemical interaction between the primary amine groups of NH2-BPSH and the dangling acyl chloride groups of polyamide [95]. In the FO operation using 40,000 ppm soybean oil/water emulsion as feed and 2 M NaCl solution as draw solution, the NH2-BPSH grafted TFC showed a high water flux of 16.5 L/m^2^·h. Water recovery up to 80% was achieved upon simple water rinsing, indicating excellent antifouling performance, which was consistent to its superhydrophilic and underwater superoleophobic properties of the modified TFC membranes. The introduction of hydrophilic NH2-BPSH oligomer was acknowledged as the main contributor of the enhanced antifouling properties as the sulfonic groups (–SO_3_), exposed on the membrane surface, reacted with water molecules to form a hydration layer that served as a barrier to prevent the attachment of oil particles in the membrane internal and surface pores. 

Li et al. investigated the performance of TFC hollow fiber membrane made from sulfonated polyphenylenesulfone (sPPSU) substrate in treating 500 ppm surfactant stabilized soy bean oil/water emulsion under FO operation [96]. The short and long term fouling studies indicated that the severe water flux decline was observed with the increasing oil concentration due to the rapid formation of oil droplet layer on the membrane surface. The permeation of the draw solution to the feed solution has also contributed to the significant flux reduction, due to the occurrence of cake-enhanced concentration polarization. The foulant layer has caused low water convection flow as a result of the increased hydraulic resistant. The accumulation of salt at the interface further worsens the concentration polarization effect and reduces the net osmotic pressure. Despite the inevitable fouling, the TFC hollow fiber membrane enabled water recovery of 80% and water flux of 10.4 L/m^2^·h while maintaining the oil rejection of more than 99%. The fouling for oil/water separation can be controlled by the addition of high pH solution of 1 g/L NaOH and 0.3 g/L sodium dodecyl sulfate (SDS) as surfactant, where the stabilized solution achieved a promising long term oil/water flux performance. TFC membrane made from electrospun polyacrylonitrile (PAN) nanofibrous substrate and polysulfone (PSf)/polyvinylpyrrolidone (PVP/cellulose acetate (CA) composite coating top layer were developed by Khamforoush et al. for oil wastewater treatment through UF process TFC membrane [97]. Compared to conventionally used asymmetric UF membranes, the flux and antifouling properties of the membrane can be more conveniently tailored by controlling the thickness of the top layer and selecting more hydrophilic polymer for the substrate layer, respectively. The addition of 1–2% CA, which acts as the hydrophilic agent, has significantly improved the water flux while the three-dimensional porosity structured of electrospun PAN has effectively reduced the likelihood of pore blockage.

**Figure 4:**
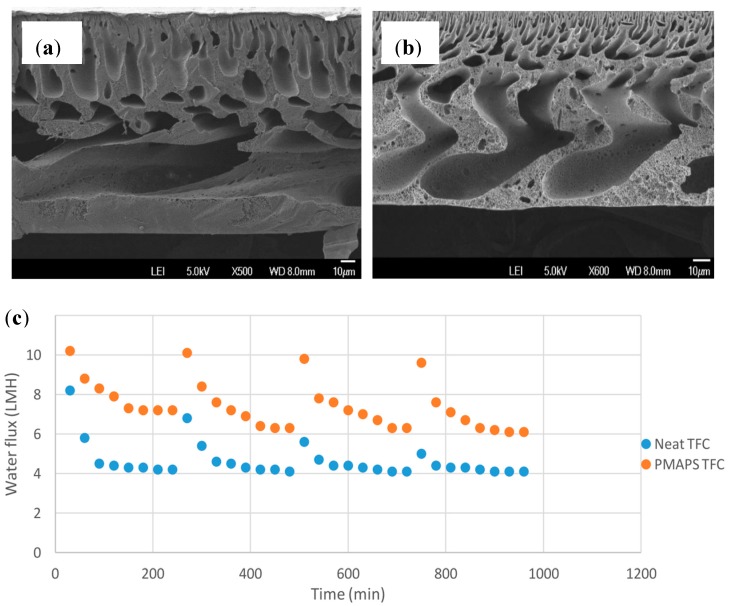
Microscopic images of (**a**) TFC membrane; (**b**) PMAPS-TFC membrane; and (**c**) Comparison of water flux for PMAPS-TFC and neat TFC membranes treated with 10,000 ppm oil emulsion over 960 min of four cycle operation, with copyright permission from [98], © 2018, Elsevier.

In a very recent study reported by Lee et al., a TFC FO membrane was prepared by incorporating PMAPS zwitterionic polymer into the polyethersulfone (PES) substrate [98]. As shown in Figure 4b, the presence of hydrophilic PMAPS has allowed the formation of well-defined finger-like structure compared to the neat TFC shown in Figure 4a. With 1 wt% of PMAPS, the TFC membrane achieved rejection of 99.9% and exhibited water flux of 15.8 L/m^2^·h, which was nearly a 30% increment compared to neat TFC. When tested using feed of 10,000 ppm oil emulsion, the modified TFC exhibited water flux recovery that are close to the initial flux upon physical rinsing with deionized water as shown in Figure 4c. The improvement was attributed to the anti-fouling properties rendered by PMAPS where the hydrogen bonding between the –SO_3_ functional groups of PMAPS and water molecule has formed a hydration layer.

Duong et al. reported the treatment of highly concentrated emulsified oily wastewater using double-skinned TFC FO membrane to produce high quality permeate [76]. The membrane consisted of polyacrylonitrile (PAN) porous substrate sandwiched by the dense polyamide layer on the top and Nexar sulfonated pentablock copolymer layer on the bottom. The selection of Nexar copolymer as the secondary layer was justified by its excellent hydrophilicity, good water permeability, high-mechanical strength, and chlorine resistance. The FO testing was performed using 0.5 M NaCl draw solution and 200,000 ppm emulsified oil as feed. Rejection of >99.9% was obtained along with a promising water flux of 10.9 L/m^2^·h. The long-term operation showed that the double-skinned TFC membrane could sustain stable flux compared to the single-skinned counterpart, which was mainly due to the anti-fouling properties of Nexar copolymer. Additionally, the fouling was also suppressed by the dense structure of the Nexar copolymer layer that has hindered the entering of emulsified oil particles into the porous support. Ong et al. fabricated double skinned membrane with the primary polyamide layer synthesized via interfacial polymerization and bottom layer grafted with PMAPS zwitterionic polymer [77]. As a result, the resultant double-skinned membrane achieved complete rejection (>99.9%) of oil rejection when tested using 10,000 ppm of emulsified oily solution as the feed and 2 M NaCl as the draw solution. The double skinned membranes membrane exhibited high water flux of 13.7 ± 0.3 L/m^2^·h, low reverse salt flux of 1.6 ± 0.2 g/m^2^·h, and high water flux recovery of up to 70%, which was with multiple-fold improvement compared to that of single-skinned TFC. The improvement was ascribed to the superior anti-fouling properties demonstrated by the hydrophilic PMAPS layer to minimize the internal fouling and ICP effects. 

**Figure 5:**
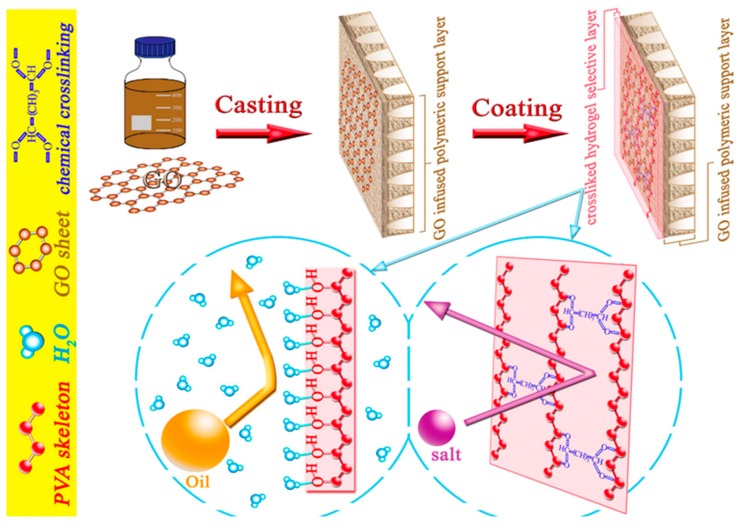
Schematic illustration of the fabrication of TFC with the thin film selective layer made of PVA hydrogels and PES substrate incorporated with GO [101].

Han et al. have developed hollow fiber TFC that consisted of porous cellulose acetate butyrate (CAB) polymeric substrate with polyamide and PDA formed on the hollow fiber outer and inner layers, respectively [99]. The double-skinned hollow fibers were used for the separation of 2000 ppm soybean oil/water emulsion via FO process using the pressure retarded osmosis (PRO), i.e., AL-DS mode using 1 M NaCl as the draw solution. Compared to CTA material, the hydrophilic CAB has provided good water permeability and is able to hamper the salt diffusivity due to the bulky hydrophobic butyrate group. The PDA coating formed a smooth and continuous thin layer to enhance surface wettability and minimize the attachment of small size oil droplets. Owing to the concerted roles played by the double skins and polymeric substrate, the resultant membrane exhibited a water flux of 37.1 L/m^2^·h with an oil rejection of 99.9%. The flux decline of 10% after operation of 12 h implied that fouling was successfully suppressed with the PDA modification. Kasemset et al. studied the effects of PDA deposition parameters on the separation and anti-fouling performances of polyamide RO membranes [100]. Commercial XLE membranes surface-modified with aqueous solution of PDA in Tris–HCl buffer were used to separate 1500 ppm emulsion of soybean oil/surfactant in water. Similarly, improvement in flux and fouling resistant was reported at the optimized PDA concentration, coating time, and pH of the buffer solution. The slight reduction in salt rejection, as compared to the unmodified RO membrane, was attributed to decrease in surface charge upon PDA coatings. Besides that, the transport properties, across the polyamide layer, were also altered by the PDA layer with lower rejection. Nevertheless, the organic rejection of more than 99.9% suggested that PDA coated XLE membrane was promising for oily wastewater treatment. Qin et al. fabricated TFC with the thin film selective layer made of polyvinyl alcohol (PVA) hydrogels and PES substrate incorporated with graphene oxide nanomaterials (GO) to treat wastewater discharged from oil and shale gas fields [101]. As shown in Figure 5, the GO nanosheet were physical mixed with PES dope prior to the phase inversion casting and subsequently the PVA hydrogel was chemically cross-linked on the substrate. During the oil/water separation, the rejection was rendered by the highly cross-linked structure of the hydrogel PVA thin film. Simultaneously, the hydrogel layer was hydrated in water to form a barrier for oil repellent and hence reduced the fouling propensity.

## 5. Challenges and Future Outlook

While extensive studies have been performed to evaluate the performance of TFC membranes for oily waste water treatment, the mechanisms of separation as well as the changes in the membrane active layer transport behavior and physicochemical properties during and after the separation processes have been scarcely reported. As the produced water contains a wide range of inorganic and organic components that may affect the membrane performance and integrity, particularly for a long term operation, the quantitative analysis and characterization are important to further assess the factors that govern the membrane performance and anti-fouling properties. As the constituents in the oily wastewater may interact with the polymeric substrate and polyamide active layer of TFC, which eventually led to membrane degradation and emergence of new organic containments in the permeate, it is certain that the better understanding of the nature and change in the membrane properties can justify the separation efficiency in terms of flux, rejection, and fouling resistance, as well as to propel the engineering advancement in this field [103]. Besides that, more reliable and standard analytical techniques are needed to accurately quantify the composition of dissolved species found in the oily wastewater, particularly produce water that has complex nature due to the presence of a wide range of contaminants such as dispersed oil, organic and inorganic compounds, heavy metal and production chemicals vary in concentration [104].

Addressing the issues related to the intrinsic limitations of membrane technology, particularly membrane fouling, is the primary aspect for practical application of this technology for oily wastewater treatment. One of the most desired efforts in this field is the development of high fouling resistant TFC membranes to improve the separation performance and enhance the sustainability of membrane separation for practical industrial application. In this respect, the fabrication of double-skinned TFC has served as a promising strategy to address this issue by introducing fouling resistant layers such as zwitterionic polymers. On the other hand, the fouling takes place in the interior of the pore walls can also be minimized through the incorporation of anti-fouling modifier within the polymeric substrate. However, despite the efforts made in the innovation of membrane design and fabrication, it is important to understand the interaction between the membrane and the oil emulsion at molecular level in order to have better insights of membrane fouling mechanism in oily waste water treatment. A more detailed understanding of the complex interaction in the colloidal system and the chemistry of oil-water-membrane interface would provide the frameworks and ideas of designing an anti-fouling membrane for this application [58]. It is also worth mentioning that an effective fouling mitigation strategy is usually achieved through the synergetic effects obtained from the combination of membrane design, module development, optimization of hydrodynamic conditions, and adaptation of cleaning protocols [105]. 

The economic factor is one of the most critical governing factors that determines the implementation of membrane technology in industries. Membrane fouling remains a main deterrent for membranes technology to be widely adopted in industries. The fundamental knowledge on the mechanisms of membrane fouling by emulsified oil is valuable to provoke innovative ideas in the design of high fouling resistant membranes. The understanding on dynamic nature of the wetting process at the water-oil-membrane interface, the effect of surface interactions on the membrane wetting, and their implications for fouling mitigation is crucial to promote the application of membrane technology for large scale oily wastewater treatment facilities. The potential application of TFC membranes for commercial-scaled oily wastewater treatment raises a critical question regarding the economic tradeoffs between lower flux asymmetric membranes, which have lower fouling propensity or higher flux TFC membranes, which has an intrinsically high fouling tendency. In the oily wastewater feed stream that is characterized by complex nature, it is clear that the physiochemical surface properties of these membranes have significant contributions to their fouling propensity. As such, it is highly desired to comprehend the interactions of membrane-oil-water interface at molecular level in order to evaluate the impacts of membrane physiochemical properties on the system performance. In view of the vulnerability of TFC surface compared to the asymmetric membrane, the justification is deemed to be a determining factor of the applicability of TFC spiral wound modules for large-scale oily wastewater treatment [106]. At the current stage, the deployment of commercial-scale FO is still largely restricted by the maintenance costs and energy consumption for draw solution recovery. Despite some efforts made in the development of cost-effective process for draw solution recovery, a significant breakthrough is yet to be made in this field [107]. While osmotically driven processes such as FO is less susceptible to fouling, the components of complex oily wastewater can result in highly potential for fouling where chemically assisted membrane cleaning is required to restore the deteriorated membrane performance. As a whole, membrane technology has been maturely applied in many commercial-scale applications. While playing a critical role in maintaining water and environmental sustainability, membrane technology itself will only become sustainable when the waste generated during the fabrication process is reduced. The considerable amount of wastewater, which was estimated as 100–500 L per square meter of membranes, generated from the manufacturing process has called for the need to adopt wastewater treatment unit in the manufacturing plants [108]. The growing environmental pollution has also prompted the exploration of green and bio-based membrane materials to tackle the issues related to the sustainability of membrane technology. Biopolymers derived from animal, vegetable sources, and bacterial fermentation products can serve as potential alternative for fossil-based polymers [109].

In the near future, the development of multi-functional TFC membrane to achieve various desired functions such as antimicrobial and photocatalytic properties can be pursued to enhance the membrane efficiency in treating oily wastewater with an increasingly more challenging nature. The recent efforts have witnessed the viability of using various modifiers such as zwitterions and PDA to impart desired properties in order to improve the intrinsic properties of TFC. In the coming years, the progress made in nanomaterial sciences is expected to open windows for more exciting modification alternatives and strategies. Additionally, the development of non-polyamide TFC membrane with comparable or better performance should also be focused on to accomplish the oily wastewater treatment. The exploration of more novel materials that possess high hydrophilicity and underwater oleophobicity in order to achieve superior fouling resistance towards various challenging natures of oil/water emulsion.

## 6. Conclusions

The establishment of economical and environmentally friendly treatment processes for oily wastewater treatment is crucial to achieve two goals, i.e., (i) to offer a source of fresh water for reuse and (ii) to meet the discharge standard and minimize serious environmental pollution. Membrane technology offers an attractive mean to efficiently treat oily wastewater to meet reuse and discharge standards. The FO process, as a resurgent membrane technology in this field, has been promoted as a promising alternative for oily wastewater treatment. While the separation using TFC membrane is currently still at the developing stage, the application of TFC membranes have effectively resolved some of the underlying issues related to asymmetric membranes used in typical UF process. The ultimate target for efficient oily wastewater treatment using FO process is to design TFC membranes that are stable, economical, and effective to treat emulsified oily wastewater in order to produce high quality reusable water and conserve scarce water resources for long-term sustainable development. Albeit the fact that a single technology is insufficient to achieve satisfactorily results for the reuse and disposal requirements for different oily wastewater, it is believed that with the continuous advancement and innovations made in the membrane design, and membrane technology can provide a versatile and economical solution that will be geared for technologically and economically sound commercial scale oily wastewater treatment.

## Figures and Tables

**Table 1:** Comparison of different membrane technologies for oil-water emulsion separation.

Membrane Technology	Advantages	Limitations	Ref.
Reverse Osmosis (RO)	Higher oil removal (>99%) e.g., treat saline oily waste Removes dissolved contaminants Higher quality effluent	High pressure requirement Membrane fouling issues caused by the trace amount of oil & grease	[24]
Forward Osmosis (FO)	Higher oil removal Low membrane fouling comparison with other pressure driven membrane processes Low or no hydraulic pressures requirement The equipment used is very simple and membrane support is less of a problem	Concentration polarization (CP) issues	[36]
Nanofiltration (NF)	Higher oil removal, large organic molecule (e.g., surfactant), hardness removal and divalent salts removal Consumes lower energy in comparison with RO processes Compact module	Higher energy consumption than FO	[37]
Microfiltration (MF)	Micron and nano-sized particulates (e.g., emulsified oil/grease) Compact modules Low energy cost No degradation due to heating No extra safety considerations as in high voltage demulsification	High energy consumption Membrane fouling of low molecular-weight MW organics	[38]
Ultrafiltration (UF)	Effective in the removal of oily microemulsions Superiority of low energy consumption and high efficiency No chemical preparation involved	Low flux Membrane fouling by property of extremely hydrophobicity	[39]

**Table 2:** Recent advances of TFC membrane for oily wastewater treatment.

TFC Substrate	TFC Active Layer	Oil/Water Flux (L/m^2^·h)	Oil Rejection (%)	Oil Concentration (mg/L)	Process	Ref.
PES/GO	PVA hydrogel	28.7	99.7	2500	FO	[101]
MATRIMID	PA	17.0	99.9	2000	FO	[102]
PES/PMAPS	PA	15.75	99.9	1000	FO	[98]
PES	PA (top) PMAPS (bottom)	<0.11	>99.9	10,000	FO	[77]
PAN	PA (top) Nexar (bottom)	<0.1893	>99.9	200,000	FO	[76]
PPSU	PA	10.4	99.62	500	FO	[96]
PAN/PSF	CA	>50	90–95	400	UF	[97]
CAB	PA (outer) PDA (inner)	37.1	99.9	2000	PRO	[99]
PSF	PA + PDA	84.0	99.9	1500	RO	[100]
